# *Amorphophallus campanulatus* tuber extract protects diabetic nephropathy in streptozotocin-induced diabetic nephropathy rat model by regulating oxidative stress and TNF-α inflammatory pathway

**DOI:** 10.1590/acb395324

**Published:** 2024-08-05

**Authors:** Li Fan, Xiaoying Li, Alok Shiomurti Tripathi

**Affiliations:** 1 Xi’an XD Group Hospital – Department of Nephrology – Xi’an, China.; 2 Air Force Medical University – The Third Affiliated Hospital – Department of Geriatrics – Xi’an, China.; 3 Era University – Era College of Pharmacy – Department of Pharmacology – Lucknow (UP) – India.

**Keywords:** Diabetic Nephropathies, Streptozotocin, Oxidative Stress, Inflammation

## Abstract

**Purpose:**

To assess the effect of *Amorphophallus campanulatus* tuber (Ac) extract in the protection of diabetic nephropathy in streptozotocin (STZ) induced diabetic nephropathy (DN) rat model.

**Methods:**

Diabetes was induced with STZ (60 mg/kg, i.p.), and DN was confirmed after six weeks of STZ administration with the estimation of kidney function test. Further rats were treated with Ac 250 and 500 mg/kg p.o. for next four week. Oxidative stress and level of inflammatory cytokines were estimated in the kidney tissue of DN rats. Histopathology of kidney tissue was performed using hematoxylin and eosin staining.

**Results:**

There was improvement in the body weight of Ac treated groups than DN group of rats. Blood glucose level was observed to be reduced in Ac treated groups than DN group on 42^nd^ and 70^th^ day of protocol. Treatment with Ac ameliorated the altered level of kidney function tests (creatinine and BUN), enzymes of liver function (aspartate aminotransferase and alanine aminotransferase), and lipid profile in the serum of DN rats. Oxidative stress parameters (malondialdehyde and reactive oxygen species enhances and reduction in the level of glutathione and superoxide dismutase) and inflammatory cytokines such as interleukin-6, tumour necrosis factor-α, and monocyte chemoattractant protein-1 reduces in the tissue of Ac treated group than DN group. Treatment with Ac also attenuates the altered histopathological changes in the kidney tissue of DN rats.

**Conclusions:**

The report suggests that Ac protects renal injury in DN rats by regulating inflammatory cytokines and oxidative stress.

## Introduction

Diabetes mellitus in long-term development of several complications causes dysfunction of different organs like impairment of kidney function known as diabetic nephropathy (DN)^1^. Prevalence of DN found to be very high between 30–40%, which is the major cause of the end-stage kidney disease throughout the globe^2^. Structural and functional abnormality of kidney are the characteristics of DN, development of it reported to be occurs due to accumulation of advanced glycation end products (AGEs), inflammation, oxidative stress, and changes in renal hemodynamic due to uncontrolled hyperglycaemia^3^^,^ 4. Production of reactive oxygen species (ROS) enhances in oxidative stress, which enhances the production of AGE, and its deposition promotes injury to kidney tissue^5^. Moreover, inflammatory cytokines such as tumour necrosis factor (TNF)-α and interleukin (IL)-6 altered the renal function, which also activates oxidative mechanism progress into DN^6^. Chemical constituents inhibit the progression of development of DN by attenuating the altered inflammatory and oxidative pathway.

Herbs are used as a complimentary medicine for the management of several chronic disorder. *Amorphophallus campanulatus* (Roxb.) Bl. belongs to Araceae family and is traditionally used for the management of tumours, piles, liver diseases, and abdominal pain. *Amorphophallus campanulatus* (AC) tuber is reported for immunomodulatory, central nervous system depressant, cytotoxicity, antifungal, antibacterial, analgesic, and antiprotease activity^7^^–^ 10. Moreover, AC has strong antidiabetic activity, which reduces blood glucose level by regulating TNF-α^11^. There are several phytochemicals such as sterols, terpenoids, coumarins, alkaloid, and flavonoids, specifically 3,5-diacetylambulin and Amblyone identified in AC tuber^12^. These phytochemicals are also known to have beneficial effects on health. Thus, the present investigation evaluated the protective effect of AC against DN.

## Methods

### Animals

Wistar rats (gender: male; age: 12 weeks old; weight: 200–230 g) were housed under controlled condition such as humidity: ~60%, temperature: 25 ± 2ºC and 12-h light/dark cycle. All the experimental protocol was approved on July 15, 2018 by the institutional animal ethical committee (650/02/C/CPCSEA/2/2014).

### Procurement and extraction Amorphophallus campanulatus

*Amorphophallus campanulatus* tubers were cut into small pieces and dried under the shade. Thereafter, we coarsely powdered it with the help of a grinder, maceration was performed by placing the powder into methanol for five days, and we shook it at a regular interval. Vacuum evaporation was performed to obtain the extract, percentage yield was found to be 11.5% w/w. Preliminary phytochemical screening revealed the presence of flavonoids, tannins, and sterols in the methanolic extract.

### Induction of diabetic nephropathy

Diabetes was induced in all the group of rats excluding control group by the single dose administration of streptozotocin (STZ) (60 mg/kg, i.p.)^13^. Blood glucose level was estimated in the serum of these rats after 72 hours of STZ administration for the confirmation of the diabetes. Glucose level more than 200 mg/dL was considered to be diabetic. All the rats were kept for six weeks, and kidney function was estimated at the end of each week for the confirmation of DN.

All the animals were grouped into four different parts – control group; DN group; AC 250 mg/kg and 500 mg/kg. The last two groups receiveed methanolic extract of AC tubers at the doses of 250 and 500 mg/kg p.o. for four weeks. Blood glucose level and body weight of each rat were estimated every week due the protocol.

### Assessment of kidney function

Kidney function was estimated by determining the biochemical level such as creatinine, BUN and lipid profile in the serum of all the groups of rats using their respective kits.

### Assessment of oxidative stress parameters

Phosphate buffer was used to homogenate the tissue sample by centrifuging it for the period of 15 min at 12,000×. Level of malondialdehyde (MDA) and activity of glutathione (GSH) and superoxide dismutase (SOD) were determined in the tissue homogenate as per the instruction of manufacturer using spectrophotometer. Moreover, level of ROS generation was also estimated as per the direction given by the manufacturer of the kit.

### Assessment of cytokine

Kidney tissue isolated from each rat was homogenized under phosphate buffer, and level of TNF-α, IL-6 and monocyte chemoattractant protein-1 (MCP-1) were estimated in the tissue homogenate of kidney of rats using enzyme-linked immunosorbent assay (ELISA) method.

### Assessment of histopathology of kidney tissue

Kidney was isolated from each animal and fixed with the help of 10% formalin solution. Further, kidney tissue was dehydrated with different concentration of ethanol and seed into molten paraffin wax to prepare wax cubes. Tissue was sectioned with the help of microtome, having 5-µm thickness, and stained with hematoxylin and eosin (H&E) staining. Assessment was performed using trinocular microscope.

### Statistical analysis

The report is represented by mean ± standard error of the mean (SEM), and GraphPad prism software version 9.3.1. (471) was used for statistical analysis with one-way analysis of variance. Significance level was considered as *p* < 0.05.

## Results

### Effect of Amorphophallus campanulatus on body weight

**Figure 1:**
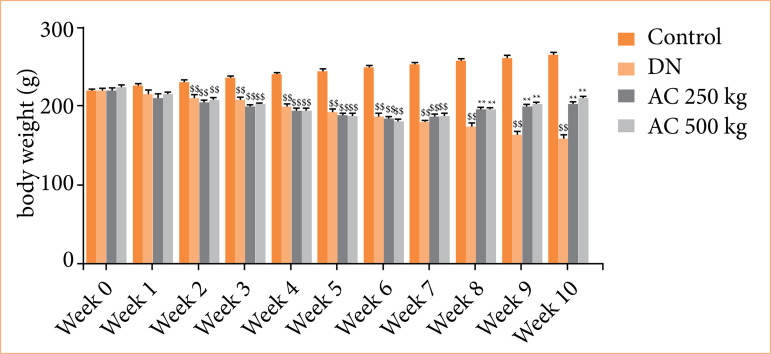
Assessment of the effect of *Amorphophallus campanulatus* on the body weight of streptozotocin induced diabetic nephropathic (DN) rats. Mean ± standard error of the mean (n = 6).

^$$^ *p* < 0.01 vs. control group; **p < 0.01 vs. DN group. Source: Elaborated by the authors.

Bodyweight was estimated in the AC treated STZ induced DN rats, as shown in Fig. 1. There was significant decrease in the body weight of STZ induced DN groups than control group for six weeks. In DN group, body weight was observed to be further reduced till the end of protocol than control group of rats. There was significant improvement in the body weight of AC treated group than DN group of rats.

### Effect of Amorphophallus campanulatus on the level of blood glucose

**Figure 2:**
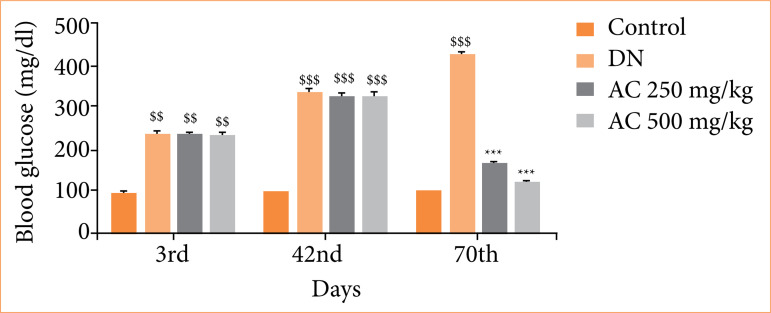
Assessment of the effect of *Amorphophallus campanulatus* on level of glucose in the serum of streptozotocin induced diabetic nephropathic (DN) rats. Mean ± standard error of the mean (n = 6).

^$$^ *p* < 0.01 vs. control group; $$$*p* < 0.001 vs. control group; ***p < 0.001 vs. DN group. Source: Elaborated by the authors.

Blood glucose level was estimated in the serum of AC treated DN rats on the third, 42^nd^ and 70^th^ day of protocol, as shown in Fig. 2. Administration STZ enhanced the level of glucose in the serum of all the groups than control group of rats on the third day of protocol, and it remained higher till the end of the 42nd day of study. Further, level of glucose enhanced in the serum of DN group than control group of rats, which was reversed in AC treated group in a dose-dependent manner.

### Effect of Amorphophallus campanulatus on the biochemical parameters

**Figure 3:**
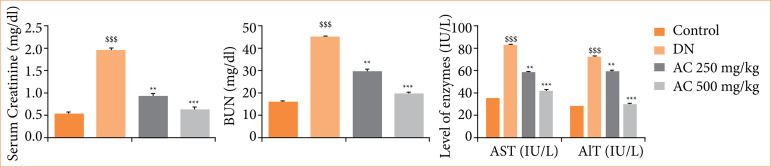
Assessment of the effect of *Amorphophallus campanulatus* on the biochemical parameters in the serum of streptozotocin induced diabetic nephropathic (DN) rats. Mean ± standard error of the mean (n = 6).

AST: aspartate aminotransferase; ALT: alanine aminotransferase; ^$$$^ *p* < 0.001 vs. control group; **p < 0.01 vs. DN group; ***p < 0.001 vs. DN group. Source: Elaborated by the authors.

Biochemical parameters were estimated in the serum of AC treated STZ induced DN rats. Levels of creatinine, BUN, aspartate aminotransferase (AST) and alanine aminotransferase (ALT) were enhanced significantly in the serum of DN group than control group. AC treated group showed significant reduction in the level of these biochemical parameters in a dose-dependent manner in the serum than DN group of rats (Fig. 3).

### Effect of Amorphophallus campanulatus on the lipid profile

**Figure 4:**
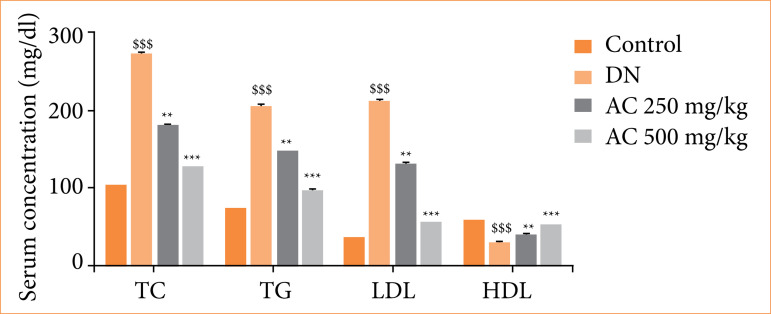
Assessment of the effect of *Amorphophallus campanulatus* on the lipid profile of streptozotocin induced diabetic nephropathic (DN) rats. Mean ± standard error of the mean (n = 6).

TC: total cholesterol; TG: triglycerides; LDL: low-density lipoprotein; HDL: high-density lipoprotein (HDL); ^$$$^ *p* < 0.001 vs. control group; **p < 0.01 vs. DN group; ***p < 0.001 vs. DN group. Source: Eaborated by the authors.

Lipid profile was observed in the serum of AC treated STZ induced DN rats. Level of total cholesterol (TC), triglycerides (TG) and low-density lipoprotein (LDL) enhanced, and reduction in the level of high-density lipoprotein (HDL) was observed in the serum of DN group than control group of rats. Treatment with AC ameliorated the altered profile of lipid in the serum of STZ induced DN rats (Fig. 4).

### Effect of Amorphophallus campanulatus on oxidative stress parameters

**Figure 5:**
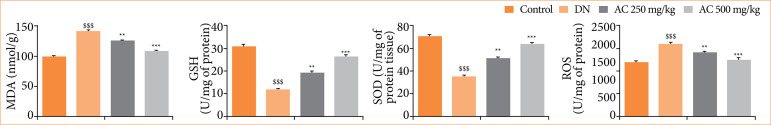
Assessment of the effect of *Amorphophallus campanulatus* on oxidative stress parameters in the kidney tissue of streptozotocin induced diabetic nephropathic (DN) rats. Mean ± standard error of the mean (n = 6).

MDA: malondialdehyde; GSH: glutathione; SOD: superoxide dismutase; ROS: reactive oxygen species; ^$$$^ *p* < 0.001 vs. control group; **p < 0.01 vs. DN group; ***p < 0.001 vs. DN group. Source: Elaborated by the authors.

Oxidative stress was estimated by determining level of its parameters in the kidney tissue of DN rats. Level of MDA and ROS enhanced, and reduction in the level of GSH and SOD were observed in the tissue of DN group than control group of rats. Treatment with AC attenuated the altered parameters of oxidative stress in the kidney tissue of DN rats (Fig. 5).

### Effect of Amorphophallus campanulatus on inflammatory cytokines

**Figure 6:**
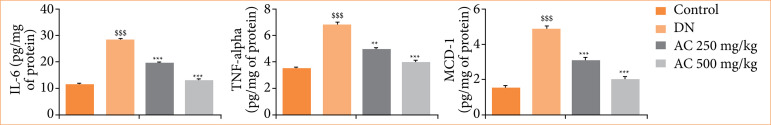
Assessment of effect of *Amorphophallus campanulatus* on mediators of inflammation in the kidney tissue of streptozotocin induced diabetic nephropathic (DN) rats. Mean ± standard error of the mean (n = 6).

IL: interleukin; TNF: tumour necrosis factor; MCP-1: MDA: malondialdehyde; GSH: glutathione; SOD: superoxide dismutase; ROS: reactive oxygen species; ^$$$^ *p* < 0.001 vs. control group; **p < 0.01 vs. DN group; ***p < 0.001 vs. DN group. Source: Elaborated by the authors.

Mediators of inflammation like IL-6, TNF-α, and MCP-1 were estimated in the kidney tissue of DN rats, as shown in Fig. 6. In DN group, level of mediators of cytokines enhanced in the kidney tissue than control group. These mediators were reduced significantly in AC treated group than DN group of rats in a dose-dependent manner.

### Effect of Amorphophallus campanulatus on histopathological changes

**Figure 7:**
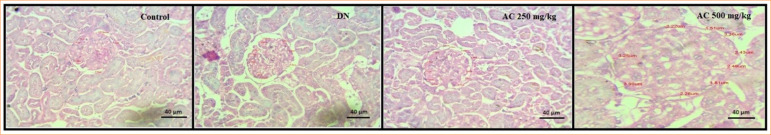
Assessment of the effect of *Amorphophallus campanulatus* on the histopathological changes in kidney tissue of streptozotocin induced diabetic nephropathic (DN) rats (n = 6).

Source: Elaborated by the authors.

Histopathological changes were estimated in the kidney tissue of AC treated DN rats by using H&E staining. Section of kidney tissue of control group showed normal appearance of morphology of glomerulus and tubules. DN group showed inflammatory changes in the kidney tissue, glomerular sclerosis, infiltration of lymphocytes, and degenerative changes in the nephrons. These changes reverse and histopathology of kidney tissue appeared to be normal in AC treated group of rats (Fig. 7).

## Discussion

Diabetes is a disorder of glucose metabolism that promotes serum concentration of blood glucose, which involves alteration of lipid profile^14^. It contributes to the enhancement of lipid level in the serum of diabetic rats, which develops atherosclerosis leading to diabetic complications including DN^13^. Clinically, patients suffering from DN shows increase in blood glucose and reduction in body weight^15^, which is supported by our study. Literature reveals that reduction in blood glucose level ceases the reduction in body weight^16^. AC is reported to possess antidiabetic activity as it reduces level of blood glucose in diabetic rats11. Data of study suggest that treatment with AC reduces the blood glucose and promotes the body weight in DN rats.

DN is one of the major chronic complications of diabetes. Several pathogenic pathways is involved in its development, including increase in lipid level that promotes injury to nephrons. Clinical study depicts the increase in creatinine and BUN level in the serum of DN patients^17^. These biochemicals are considered as the markers of renal failure, and an increase in the level of these markers deemed to be clinical feature of renal injury. Reduction in the level of these biochemical parameters of kidney function in the serum mean the protection of renal injury^18^, which was supported by this study, as AC treated group showed reduced level of BUN and creatinine in the serum of DN rats.

Lipid profile alters in DN, as LDL, TC and TG level enhances and HDL level reduces in DN. This altered level of lipid profile, specifically increased levels of LDL, promotes oxidative stress in the nephron tissue^19^. MDA is one of the major markers of oxidative stress, known to enhance its level in nephropathy. Production of ROS enhances in due to alteration in the lipid level, which promotes the production of superoxide anions^20^. These superoxide anions failed to be digested by SOD, leading to alterations in the integrity of cell membrane. MDA is a content of cell membrane due to injury to it, and the release of MDA in the serum causes increase in the level of MDA. These changes could appear due to the reduction in the level of GSH and SOD and increase in production of ROS^21^. The study revealed that treatment with AC ameliorates the altered lipid profile and oxidative stress parameters in the serum and kidney tissue homogenate of DN rats, respectively. These changes in lipid profile and oxidative stress of AC shows the protective effect of it against DN.

Damage to the cell membrane of nephrons activates the inflammatory pathway, as it promotes infiltration of lymphocytes, which further contributes in the development of injury to nephrons^22^. Inflammatory mediators like TNF-α and IL-6 are the majorly cytokines involved in injury to nephrons, and infiltration of lymphocytes due to increase in MCP-1 and regulation in the level of these mediators promotes injury to nephrons^23^. Data of given report suggest that treatment with AC reduces the level of these mediators to protect the renal injury in DN rats. Moreover, histopathology study also revealed that treatment with AC reverses the histopathological changes in the kidney tissue of DN rats.

## Conclusion

The present study shows the protective effect of AC against DN, as it regulates level of oxidative stress and inflammatory pathway in STZ induced DN rats. Results shows AC could be used clinically for the management of DN.

## Data Availability

All data generated or analyzed during this study are included in this article.
